# Supplementing tuberculosis surveillance with automated data from health maintenance organizations.

**DOI:** 10.3201/eid0506.9906

**Published:** 1999

**Authors:** D S Yokoe, G S Subramanyan, E Nardell, S Sharnprapai, E McCray, R Platt

**Affiliations:** Brigham and Women's Hospital, Boston, Massachusetts, USA. yokoe@channing.harvard.edu

## Abstract

Data collected by health maintenance organizations (HMOs), which provide care for an increasing number of persons with tuberculosis (TB), may be used to complement traditional TB surveillance. We evaluated the ability of HMO-based surveillance to contribute to overall TB reporting through the use of routinely collected automated data for approximately 350,000 HMO members. During approximately 1.5 million person-years, 45 incident cases were identified in either HMO or public health department records. Eight (18%) confirmed cases had not been identified by the public health department. The most useful screening criterion (sensitivity of 89% and predictive value positive of 30%) was dispensing of two or more TB drugs. Pharmacy dispensing information routinely collected by many HMOs appears to be a useful adjunct to traditional TB surveillance, particularly for identifying cases without positive microbiologic results that may be missed by traditional public health surveillance methods.


### Supplementing Tuberculosis Surveillance with Automated Data from Health Maintenance Organizations

**Deborah S. Yokoe,* Girish S. Subramanyan,* Edward Nardell,† Sharon Sharnprapai,† Eugene McCray,‡ and Richard Platt*§**
*Brigham and Women's Hospital, Boston, Massachusetts, USA; †Massachusetts Department of Public Health, Jamaica Plain, Massachusetts, USA; ‡Centers for Disease Control and Prevention, Atlanta, Georgia, USA; and §Harvard Medical School, Harvard Pilgrim Health Care, Boston, Massachusetts, USA

Data collected by health maintenance organizations (HMOs), which provide care for an increasing number of persons with tuberculosis (TB), may be used to complement traditional TB surveillance. We evaluated the ability of HMO-based surveillance to contribute to overall TB reporting through the use of routinely collected automated data for approximately 350,000 HMO members. During approximately 1.5 million person-years, 45 incident cases were identified in either HMO or public health department records. Eight (18%) confirmed cases had not been identified by the public health department. The most useful screening criterion (sensitivity of 89% and predictive value positive of 30%) was dispensing of two or more TB drugs. Pharmacy dispensing information routinely collected by many HMOs appears to be a useful adjunct to traditional TB surveillance, particularly for identifying cases without positive microbiologic results that may be missed by traditional public health surveillance methods.

As more persons move into managed health-care organizations, traditional tuberculosis (TB) surveillance methods, which rely heavily on information collected and channeled through the public health system, may need to be supplemented. Accurate, complete surveillance information is important for identification and follow-up of persons with TB, as well as for accurate assessment of the impact of TB on public health, the effectiveness of control activities, and the planning and prioritizing of interventions.

Automated data routinely collected by managed care organizations may complement TB surveillance obtained through reporting to local and state health departments. In this study, we evaluated the use of pharmacy dispensing information and other inpatient and ambulatory-patient data routinely collected by managed care organizations for identifying TB cases.

Address for correspondence: Deborah S. Yokoe, 181 Longwood Ave., Boston, MA 02115, USA; fax: 617-731-1541; e-mail: deborah.yokoe@channing.harvard.edu.

### Methods

### Study Population

The study population consisted of approximately 350,000 persons with pharmacy coverage who received their care at one of the 14 Harvard Pilgrim Health Care centers with automated full-text medical records for ambulatory patients and 100,000 persons with pharmacy coverage at 17 practices without such records within Massachusetts from January 1, 1992, to June 30, 1996. Automated pharmacy and billing data, however, were available for the entire study population.

### Identification of TB Cases from HMO Records

Ambulatory care, hospital, and emergency room claims for the entire study population were screened for any of 60 International Classification of Diseases,, 9th Revision Clinical Modification diagnosis codes or current procedures terminology codes suggestive of TB. Automated pharmacy records were searched for dispensing of any of 10 antituberculosis medications during the study period (Table 1). The automated ambulatory-patient record, available for approximately 250,000 persons within our study population, has been described in detail (1). The automated medical record system uses standardized forms that are completed for every patient encounter at specific Harvard Pilgrim Health Care centers, including telephone calls, office visits, urgent care visits, and hospitalizations. For each encounter, the provider either writes in or selects from a list of all coded diagnoses, tests, procedures, and prescriptions and enters additional comments as free text. The automated ambulatory-patient records were also screened

Table 1. Components of the health maintenance organization-based screening criteria for tuberculosis (TB)

| Code type | Code | Description of code |
|---|---|---|
| **Antituberculosis drugs** | | |
| Pharmacy dispensing | | Isoniazid |
| Pharmacy dispensing | | Ethambutol |
| Pharmacy dispensing | | Rifampin |
| Pharmacy dispensing | | Pyrazinamide |
| Pharmacy dispensing | | Streptomycin |
| Pharmacy dispensing | | Para-aminosalicyclic acid (PAS) |
| Pharmacy dispensing | | Kanamycin |
| Pharmacy dispensing | | Capreomycin |
| Pharmacy dispensing | | Cycloserine |
| Pharmacy dispensing | | Ethionamide |
| **Microbiology codes** | | |
| CPT[a] | 87015 | Concentration (any type) for parasites, ova, or tubercle bacillus (TB, AFB) |
| CPT | 87116 | Culture, tubercle, or other acid-fast bacilli; any source, isolation only |
| CPT | 87117 | Culture, tubercle, or other acid-fast bacilli; concentration plus isolation |
| CPT | 87118 | Culture, mycobacteria, definite identification of each organism |
| CPT | 87190 | Sensitivity studies, antibiotic; tubercle bacillus (TB, AFB), each drug |
| CPT | 87206 | Smear, primary source, with interpretation; fluorescent or acid-fast stain for bacteria, fungi, or cell types |
| ICD-9[b] procedure | 90.4 | Microscopy examination of sputum |
| ICD-9 procedure | 90.41 | Bacterial smear |
| ICD-9 procedure | 90.42 | Culture |
| ICD-9 procedure | 90.43 | Culture and sensitivity |
| ICD-9 procedure | 90.49 | Other microscopic examination |
| COSTAR[c] | TB234 | AFB smear |
| COSTAR | TB850 | AFB culture and sensitivity |
| **Radiology codes** | | |
| CPT | 71010 | Chest, single view, frontal |
| CPT | 71020 | Chest, two views, frontal and lateral |
| CPT | 71021 | Chest with apical lordotic procedure |
| CPT | 71030 | Chest, complete, minimum of four views |
| CPT | 71250 | CT, thorax, without contrast |
| CPT | 71260 | CT, thorax, with contrast |
| CPT | 71270 | CT, thorax, without contrast, followed by contrast |
| CPT | 71550 | MRI[d] chest |
| CPT | 71555 | MRI chest (excluding myocardium) |
| ICD-9 procedure | 87.44 | Chest X-ray |
| COSTAR | TR027 | Chest, PA[e] only |
| COSTAR | TR028 | Chest X-ray |
| COSTAR | TR029 | Chest, PA, and last with fluoroscopy |
| COSTAR | TR032 | Chest, fluoroscopy |
| COSTAR | TR178 | MRI-chest |
| COSTAR | TR184 | CAT[f] scan-chest |
| COSTAR | TR236 | Chest, PA, and lateral |
| COSTAR | TR237 | Chest-PA, lateral, both obliques |
| COSTAR | TR238 | Chest-four views |
| COSTAR | TR240 | Chest-special views |

[a]CPT, current procedures terminology.
[b]ICD9, International Classification of Diseases, 9th revision.
[c]COSTAR, coding system used for the automated ambulatory-patient medical records (10).
[d]MRI, magnetic resonance imaging.
[e]PA, posteroanterior.
[f]CAT, computer-assisted tomography.
[g]PPD, purified protein derivative of tuberculin.

Table 1, cont'd. Components of the health maintenance organization-based screening criteria for tuberculosis (TB)

| Code type | Code | Description of code |
|---|---|---|
| **PPD[g] status** | | |
| COSTAR | DG249 | Positive PPD |
| COSTAR | DA129 | Tuberculin conversion |
| **Diagnosis codes for TB** | | |
| COSTAR | DR185 | TB |
| COSTAR | DG250 | Pulmonary TB |
| COSTAR | DG251 | Active TB |
| ICD-9 diagnosis | 010.0 | Primary TB infection |
| | 010.1 | |
| | 010.8 | |
| ICD-9 diagnosis | 011.0 | Pulmonary TB |
| | 011.1 | |
| | 011.2 | |
| | 011.3 | |
| | 011.5 | |
| | 011.6 | |
| | 011.8 | |
| | 011.9, | |
| | 011.90-011.96 | |
| ICD-9 diagnosis | 012.0 | Other respiratory TB |
| | 012.1 | |
| | 012.2 | |
| ICD-9 diagnosis | 013.0 | TB of meninges and central nervous system |
| | 013.1 | |
| | 013.2 | |
| | 013.3 | |
| | 013.4 | |
| | 013.5 | |
| ICD-9 diagnosis | 015.0 | TB of bones and joints |
| | 015.7 | |
| | 015.8 | |
| | 015.9 | |
| ICD-9 diagnosis | 016.0 | TB of genitourinary system |
| | 016.3 | |
| ICD-9 diagnosis | 017.2 | TB of peripheral lymph nodes |
| ICD-9 diagnosis | 018.0 | Miliary TB |
| | 018.8 | |
| | 018.9 | |
| ICD-9 diagnosis | 795.3 | Sputum positive only |
| **Bronchoscopy and biopsy** | | |
| ICD-9 procedure | 33.22-33.24 | Diagnostic procedures on lung and bronchus |
| | 33.26-33.28 | Biopsy of lymphatic structure |
| | 40.11 | |

[a]CPT, current procedures terminology.
[b]ICD9, International Classification of Diseases, 9th revision.
[c]COSTAR, coding system used for the automated ambulatory-patient medical records (10).
[d]MRI, magnetic resonance imaging.
[e]PA, posteroanterior.
[f]CAT, computer-assisted tomography.
[g]PPD, purified protein derivative of tuberculin.

for any one of 17 coded diagnoses, tests, and procedures suggestive of TB (Table 1).

Twelve combinations of screening codes suggestive of active TB were used for automated ambulatory-patient records, and five combinations of screening codes were used for other records (Table 2). To limit the number of persons meeting screening criteria, we focused on combinations of codes likely to have the highest yield of TB cases. Cases that met any of these screening criteria were assessed further.

Full-text ambulatory-patient medical records were reviewed for all persons identified by screening criteria who had automated ambulatory records. For individuals identified through screening who did not have automated ambulatory records, a modified version of the Centers for Disease Control and Prevention's

Table 2. Performance of health maintenance organization-based screening criteria for tuberculosis (TB)

| Screening criteria | No. meeting screening criteria | No. TB cases detected using screening criteria | No. TB cases unknown to public health dept. | Sensitivity (95% CI) | Positive predictive value (95% CI) |
|---|---|---|---|---|---|
| **All patients (45 incident TB cases)** | | | | | |
| Two or more anti-TB drugs[a] | 133 | 40 | 7 | 89 (76,96) | 30 (22, 39) |
| Two or more anti-TB drugs[a] dispensed on the same date | 108 | 39 | 7 | 87 (73,95) | 36 (27,50) |
| Three or more anti-TB drugs[a] | 76 | 38 | 7 | 84 (71,94) | 50 (38,62) |
| **Only patients with automated medical records (41 incident TB cases)** | | | | | |
| One or more anti-TB drugs,[a] a microbiology code,[b] and a radiology code[c] | 132 | 21 | 2 | 51 (35, 67) | 16 (10, 23) |
| At least one anti-TB drug[a] and a CPT[c] code for mycobacterial culture/stain | 106 | 17 | 2 | 42 (26,58) | 16 (10,24) |
| Diagnosis code[d] for tuberculosis, a microbiology code,[b] and a radiology code[c] | 49 | 16 | 0 | 39 (24,56) | 33 (20,48) |
| Diagnosis code[d] for positive PPD,[e] a microbiology code,[b] and a radiology code[c] | 157 | 8 | 1 | 20 (9,35) | 5 (2,10) |
| At least one anti-TB drug[a] and an ICD-9 diagnosis code for tuberculosis | 14 | 7 | 1 | 17 (7,32) | 50 (23,77) |
| ICD-9 procedure code for bronchoscopy, a microbiology code,[b] and a radiology code[c] | 15 | 1 | 0 | 2 (0.1,13) | 7 (0.2,32) |
| Diagnosis code[d] for active tuberculosis | 4 | 1 | 0 | 2 (0.1,13) | 25 (1,81) |
| Diagnosis code[d] for pulmonary tuberculosis | 75 | 0 | 0 | 0 | 0 |
| Diagnosis code[d] for tuberculin conversion, a microbiology code,[b] and a radiology code[c] | 1 | 0 | 0 | 0 | 0 |
| **Only patients without automated medical records (4 incident TB cases)** | | | | | |
| ICD-9 diagnosis code for tuberculosis | 251 | 4 | 2 | 100 (40, 100) | 2 (0.4, 40) |
| A CPT code relating to mycobacterial culture/stain or a radiology code | 92 | 2 | 1 | 50 (7, 93) | 2 (0.3, 8) |

[a]Pharmacy dispensing data; antituberculosis drugs include isoniazid, rifampin, pyrazinamide, ethambutol, streptomycin, capreomycin, kanamycin, ethionamide, para-aminosalicyclic acid, and cycloserine.

[b]Microbiology codes include COSTAR (coding system for automated ambulatory-patient records [10]) or ICD-9CM (International Classification of Diseases, 9th Revision Clinical Modification) procedure codes for acid fast bacilli smear, culture and sensitivities and microscopy examination of sputum.

[c]Radiology codes include current procedures terminology (CPT), COSTAR, or ICD-9 procedure codes for chest radiograph, thoracic computer assisted tomography (CT), or thoracic magnetic resonance imaging (MRI).

[d]Ambulatory codes were obtained from automated ambulatory-patient records in the staff model division and from claims in the network and group model division.

[e]PPD, purified protein derivative.

(CDC) Report of Verified Case of Tuberculosis form was sent to the primary-care physicians. The form is routinely used to report to CDC individual TB case information, including clinical characteristics and laboratory results. Our modified form included the question “While under your care, did this patient have suspected and/or confirmed ACTIVE tuberculosis?” If “Yes” was checked, the full-text medical records of the person were reviewed. In addition, the medical records of a random sample of 10% of the patients with questionnaires returned by providers were reviewed to validate the use of data obtained from questionnaire results. A case of TB was defined according to the CDC surveillance definition (2). A culture-positive case is defined as isolation of *Mycobacterium tuberculosis* from a clinical specimen. A smear-positive case is defined as demonstration of acid-fast bacilli (AFB) in a specimen if either a culture was not obtained or results were unknown. In the absence of laboratory evidence of disease, a clinical case is one that meets the following criteria: a positive tuberculin skin test, a completed diagnostic work-up, clinical evidence and signs and symptoms compatible with TB, an abnormal and unstable (worsening or improving) chest radiograph if intrathoracic disease is present, and treatment with two or more antituberculosis drugs. All cases without a positive culture for *M. tuberculosis* that were not known to the public health department were verified by review with the Massachusetts State Tuberculosis Control Officer, using all available primary patient data from the ambulatory-patient medical record, public health records, and hospital records.

### Identification of TB Cases from Public Health Department Records

Reporting of confirmed or clinically suspected TB cases to the Massachusetts Department of Public Health by health-care providers, laboratories, boards of health, or administrators of hospitals is mandatory. In addition, the Massachusetts State Laboratory Institute performs susceptibility testing on most *M. tuberculosis* isolates in Massachusetts and provides the public health department with direct access to microbiology information about virtually all persons in Massachusetts with culture-positive *M. tuberculosis*. All verified cases are entered into the public health TB registry.

The entire HMO population was matched to the public health TB registry by using limited patient identifiers (first two letters of last name, first two letters of first name, month and year of birth, and sex) to maintain patient confidentiality. Potential matches were confirmed by using full identifiers. This method for matching registries with minimal disclosure of individual identities is described elsewhere (3).

### Analysis

The sensitivity, defined as the proportion of TB cases detected by either HMO-based screening criteria or routine public health surveillance, was determined by comparison with any verified TB case identified through public health or HMO records. Positive predictive value was defined as the proportion of persons with verified TB meeting screening criteria. Exact binomial confidence intervals were calculated for sensitivity and positive predictive value (4). The performance of the different screening rules for detecting TB was compared.

### Results

In approximately 1.5 million person-years, 768 persons met at least one of the HMO-based screening criteria, with a positive screening criteria rate of 0.4 per 10,000 person-years among persons with automated ambulatory-patient records and 0.7 per 10,000 person-years among those without such records. Thirty-nine (9%) incident TB cases were identified among the 415 persons with automated ambulatory-patient records who met screening criteria, and 4 (1%) incident TB cases were identified among the 353 persons without automated ambulatory records who met screening criteria. The response rate to the provider questionnaire was 100%, as was the agreement rate between classification of TB cases based on provider questionnaire results and on-site medical record review.

Thirty-five (81%) of the 43 incident TB cases detected by HMO-based screening had been identified previously by the public health department. Of these 35 cases, 32 were culture-positive, and three met the clinical case definition. Two additional TB cases, both of which were culture-positive, were known to the public health department but did not meet HMO-based screening criteria. These two patients received treatment and medication from state-funded TB clinics. Thus, 45 cases were identified through either HMO-based screening or public health department records. All 45 cases met the CDC surveillance definition. Eight (18%) of these cases were unknown to the public health system. Most cases (41 of 45) were diagnosed at one of the HMO centers with automated ambulatory-patient records, a proportion consistent with the concentration of urban regions within their catchment areas. The rates were approximately 11.7 TB cases per 100,000 population among HMO members with automated ambulatory-patient records and four TB cases per 100,000 population among those without such records.

The sensitivity of each of the screening criteria was 0% to 100%, and the positive predictive value was 0% to 52% (Table 2). Screening criteria based on pharmacy dispensing information had the best combinations of sensitivity and positive predictive value. Two or more dispensed antituberculosis drugs, combining the results for persons with and without automated ambulatory-patient records, had an overall sensitivity of 89% (95% confidence interval [CI] = 76%, 96%) and positive predictive value of 30% (95% CI = 22%, 39%). Three or more antituberculosis drugs had an overall sensitivity of 84% (95% CI = 71%, 94%) and positive predictive value of 50% (95% CI = 38%, 62%), and two or more antituberculosis drugs dispensed on the same date had an overall sensitivity of 87% (95% CI = 73%, 95%) and positive predictive value of 36% (95% CI = 27%, 50%). The differences between the performance of two or more dispensed antituberculosis drugs among persons with automated ambulatory-patient records (sensitivity = 90%, positive predictive value = 34%) and persons without automated records (sensitivity = 75%, positive predictive value = 12%) were not statistically significant, although the small number of TB cases in each group limits the power to detect a difference.

Among the 71 persons with automated ambulatory-patient records who received two or more antituberculosis drugs but did not have incident TB, 9 (13%) had active TB diagnosed outside the study period, 23 (32%) were treated for other mycobacterial infections, 11 (15%) received more than one drug during TB prophylaxis, 2 (3%) received drugs for multiple unrelated conditions (e.g., rifampin for eradication of *Staphylococcus aureus*; ethambutol for *M. avium* complex prophylaxis), and the remaining 26 (37%) were suspected of having active TB without subsequent confirmation (Table 3).

Of the 118 persons with automated ambulatory-patient records who met screening criteria involving a diagnosis code for TB but did not have incident TB, 57 received the diagnosis code as an indication of routine prenatal screening for TB, 12 had a previous history of TB, 31 were suspected of having active TB without subsequent confirmation, and 18 had the diagnosis code documented in their HMO ambulatory medical record for no apparent reason (Table 3).

Of the eight patients whose cases had not been identified by the public health department, seven were culture-negative and met the TB clinical case definition, and one did not have a microbiology culture and met the smear-positive TB case definition. Three of the patients had AFB smear-positive pathology specimens; of these, two had negative cultures for *M. tuberculosis,* and one did not have a culture performed. Of the eight cases, one involved pulmonary TB, and the remaining seven were extrapulmonary. One patient was 2 years old at the time of diagnosis; the remaining seven were 18 years of age or older. All cases were confirmed by review with the Massachusetts State Tuberculosis Control Officer. Of these eight cases, seven were detected by the two or more antituberculosis drug screening criterion.

### Conclusions

Since the establishment of a national surveillance system for TB in 1953, TB surveillance has depended on laboratories, public health clinics, and reporting by private practitioners. Several retrospective studies performed by local TB programs suggest that TB cases may be underreported (5-7). Although ascertainment of culture-positive cases is likely to be nearly complete, since laboratories are required by law in most states to report isolation of *M. tuberculosis* to the state health department, surveillance for cases lacking positive cultures depends largely on reporting by health-care providers or referrals to public health clinics for treatment. Underreporting of TB cases without positive cultures may contribute to incomplete surveillance. A study assessing the completeness of TB case reporting

Table 3. Reasons for meeting screening criteria among individuals without incident tuberculosis (TB) who had automated ambulatory-patient records

| Reasons why non-TB cases met screening criteria | Screening criteria that include a TB diagnosis code or multiple anti-TB drugs: Two or more anti-TB drugs[a] | Diagnosis code[b] for active TB | Diagnosis code[b] for pulmonary TB | Diagnosis code[b] for TB, a microbiology code,[c] and a a radiology code[d] | At least one anti-TB drug[a] and an ICD-9 diagnosis code for TB |
|---|---|---|---|---|---|
| Active TB diagnosed outside study window | 9 (13%) | 0 | 0 | 0 | 0 |
| Suspected active TB | 26 (37%) | 2 (67%) | 4 (5%) | 19 (58%) | 6 (86%) |
| TB prophylaxis | 11 (15%) | 0 | 0 | 0 | 0 |
| Prenatal TB screening | 0 | 0 | 57 (76%) | 0 | 0 |
| Prior history of TB | 0 | 0 | 5 (7%) | 7 (21%) | 0 |
| Other mycobacterial infections | 23 (32%) | 0 | 0 | 0 | 0 |
| Treatment of other conditions | 2 (3%) | 0 | 0 | 0 | 0 |
| No documentation of reason in HMO medical record | 0 | 1 (33%) | 9 (12%) | 7 (21%) | 1 (14%) |
| Total no. without incident active TB | 71 | 3 | 75 | 33 | 7 |

[a]Pharmacy dispensing data; antituberculosis drugs include isoniazid, rifampin, pyrazinamide, ethambutol, streptomycin, capreomycin, kanamycin, ethionamide, para-aminosalicyclic acid, and cycloserine.
[b]Ambulatory codes were obtained from automated ambulatory records in the staff model division and from claims in the network and group model division.
[c]Microbiology codes include COSTAR (coding system for automated ambulatory-patient records [10]) or ICD-9CM (International Classification of Diseases, 9th Revision Clinical Modification) procedure codes for acid fast bacilli smear, culture and sensitivities and microscopy examination of sputum.
[d]Radiology codes include current procedures terminology (CPT), COSTAR, or ICD-9 procedure codes for chest radiograph, thoracic computer- assisted tomography (CT), or thoracic magnetic resonance imaging (MRI).

in Puerto Rico (6) found that 19.5% of patients with TB were not reported, partly because of underreporting of cases without positive cultures for *M. tuberculosis*.

The recent shift into managed care of populations at high risk for TB, including Medicaid and Medicare recipients, has raised additional concern about the continued completeness of reporting. However, HMOs routinely collect information that can be used to identify persons likely to have TB. McCray et al. (6) noted that, according to pharmacy prescription data in Maryland, the cases of 19% of patients receiving two or more antituberculosis drugs had not been reported to the public health department; however, the patients' medical records were not reviewed to verify a diagnosis of active TB. Maggini et al. (8) evaluated the use of Italy's National Health Service pharmacy dispensing information to identify TB cases in the province of Rome and found that pharmacy screening detected seven times more new TB cases than routine passive surveillance. Hripcsak et al. (9) evaluated a number of screening rules based on automated information available at an urban medical center in New York City and found that inpatient use of antituberculosis drugs had a sensitivity of 68% and a positive predictive value of <1% for detecting TB cases based on their health department's TB registry. These investigators did not, however, have access to records of antituberculosis drugs received by ambulatory patients and did not specifically evaluate the use of more than one antituberculosis drug as a screening criterion. No previous study has compared the utility of pharmacy data with that of other automated administrative or health-care data.

Of the screening criteria evaluated in our study, dispensing of two or more antituberculosis drugs was the most useful, with an overall sensitivity of 89%. The most common reasons for dispensing of two or more antituberculosis drugs to persons without TB were the empiric use of antituberculosis drugs for suspected active TB (37%) and the use of antituberculosis drugs for treatment of mycobacterial infections other than TB (32%). In addition, 15% of patients without TB received more than one drug for TB prophylaxis, which can occur, for example, when isoniazid is switched to another antituberculosis drug because of adverse drug reactions. A possible strategy for improving the positive predictive value of screening criteria based on pharmacy dispensing information is the use of more rigorous criteria, such as dispensing of three or more antituberculosis drugs (positive predictive value = 50%), or restricting the timing of drug dispensing, such as requiring that two or more antituberculosis drugs be dispensed on the same date (positive predictive value = 36%). The improvement in positive predictive value for these more rigorous criteria, however, must be weighed against loss of sensitivity in identifying TB cases. For our HMO study population, requiring three or more antituberculosis drugs missed two TB cases, and requiring that two or more drugs be dispensed on the same date missed one case detected by the less stringent criterion. The choice of the screening criterion with the most useful balance between sensitivity and specificity depends in part on the surveillance strategy used.

Surveillance based on HMO pharmacy dispensing information can be used to identify HMO enrollees most likely to have active TB, so that efforts can be focused on additional evaluation of these persons. As with traditional TB surveillance methods, surveillance based on pharmacy dispensing information requires information from the patients' medical records to verify whether the TB case definition is satisfied. Using this surveillance strategy, screening for two or more antituberculosis drugs would require reviewing the medical records of approximately three patients to identify each case of incident active TB. We feel that the positive predictive value of 30% is sufficient to make this surveillance screening method practical if it can be applied in other managed care settings.

The positive predictive values of screening criteria that include TB diagnosis codes are limited by a number of factors. TB diagnosis codes, for example, were frequently used for patients with suspected active TB during the weeks required for diagnostic work-up or observation for clinical response to therapy. These codes were also frequently used to indicate that routine TB skin testing had been performed rather than to indicate the presence of active disease or prior history of TB.

The difference in the TB case rates between HMO members with automated ambulatory-patient records (approximately 11.7 TB cases per 100,000 population) and members without such records (approximately four TB cases per 100,000 population) in our study could either reflect a true difference in the underlying risk for TB in the two populations or case ascertainment bias resulting from differences in the methods used to identify TB cases. The former explanation is more likely for several reasons. First, the HMO health centers with automated ambulatory-patient records serve a largely urban population concentrated in the Boston area, while the HMO-affiliated practices without such records serve a largely suburban population. The difference in the rates found in our study mirrors the difference in the 1992 to 1998 TB case rate averages reported by the Massachusetts Department of Public Health for the city of Boston (17.7 TB cases per 100,000 population) compared with the rest of the state of Massachusetts (4.1 TB cases per 100,000 population). Second, the match between the health department's TB registry and the HMO membership list did not identify any TB patients who had not previously been detected through screening criteria and record review based on our modified RVCT results among HMO members without automated ambulatory patient records. This argues against inadequate case finding resulting in apparent lower TB case rates in this group.

A substantial number of TB cases in our study were unknown to the public health department (18% of cases among our HMO study population). This proportion is comparable with the fraction described in the studies cited above. Underreporting of these cases compromises the usefulness of TB surveillance. Screening for dispensing of antituberculosis drugs may be a particularly useful method for identifying cases without positive cultures for *M. tuberculosis* that might otherwise be missed by routine surveillance methods dependent on laboratory- and provider-based reporting.

The positive predictive value of screening criteria based on the dispensing of antituberculosis drugs may also be limited to some degree in clinical settings where many patients receive these medications for other indications, including other mycobacterial infections (e.g., in cases of HIV infection). Strategies that could be applied in such settings include excluding those persons also receiving medications frequently used for treatment of *M. avium* complex infections (e.g., clarithromycin). During our study period, however, more than 1,000 known HIV-infected patients were treated in HMO centers with automated ambulatory-patient records, of whom only 23 (Table 3) had false-positive cases identified by the two or more TB drug criterion. In addition, widespread implementation of new CDC recommendations for use of multidrug therapy for TB prophylaxis may require modification of the screening criteria. One possible strategy would be to require that antituberculosis drugs be dispensed over a minimum time interval (e.g., >4 months).

Although TB surveillance based on pharmacy dispensing information depends upon availability of automated pharmacy data, such data are available for most of the U.S. population, including most Medicaid and Medicare recipients. Our results indicate that pharmacy dispensing information routinely collected by many HMOs has high sensitivity and reasonable positive predictive value and is particularly useful for identifying TB cases without positive cultures, which may be missed by traditional public health surveillance.

### Acknowledgments

We thank Matthew McKenna for his contributions to the planning of this study, Liz Martino and Ralph Blair for their help in evaluating the Harvard Vanguard data, and Claire Canning, Linda Lacke, and Shirley Golberg for their assistance in this study.

Supported by the American Association of Health Plans and CDC Contract 200-95-0957-010.

Dr. Yokoe is an associate physician at Brigham and Women's Hospital in Boston, Massachusetts, and an instructor in Medicine at Harvard Medical School.

### References

1. Platt R. Studies of prescription drugs at Harvard Community Health Plan. In: Strom B, editor. Pharmacoepidemiology. 2d ed. New York: John Wiley and Sons; 1994. p. 278-87.
2. Centers for Disease Control and Prevention. Case definitions for infectious conditions under public health surveillance. MMWR Morb Mortal Wkly Rep 1997;46(RR-10):40-1.
3. Subramanyan G, Yokoe D, Sharnprapai S, Tang Y, Platt R. An algorithm to match registries with minimal disclosure of individual identities. Public Health Reports 1999;114:91-3.
4. Stata statistics and data analysis [computer program]. Version 5.0. College Station (TX): Stata Corporation; 1997.
5. Marier R. The reporting of communicable diseases. Am J Epidemiol 1977;105:587-90.
6. McCray E, Weinbaum CM, Braden CR, Onorato IM. The epidemiology of tuberculosis in the United States. Clin Chest Med 1997;18:99-113.
7. Driver C, Braden CR, Nieves R, Navarro AM, Rullan JV, Valway SE, et al. Completeness of tuberculosis case reporting, San Juan and Caguas Regions, Puerto Rico, 1992. Public Health Rep 1996;111:157-61.
8. Maggini M, Salmaso S, Alegiani SS, Caffari B, Raschetti R. Epidemiological use of drug prescriptions as markers of disease frequency: An Italian experience. J Clin Epidemiol 1991;44:1299-307.
9. Hripcsak G, Knirsch C, Jain N, Pablos-Mendez A. Automated tuberculosis detection. JAMIA 1997;4:376-81.
10. Winickoff RN, Barnett GO, Morgan M, Coltin KL. Quality assurance in a prepaid group practice. Journal of Ambulatory Care Management 1979;2:19-28.
